# Can Clean Delivery Kits Prevent Infections? Lessons from Traditional Birth Attendants in Nigeria

**DOI:** 10.5334/aogh.4015

**Published:** 2023-12-06

**Authors:** Adediwura Oladunni Arowosegbe, Iyabode Olabisi Dedeke, Olufunke Bolatito Shittu, David Ajiboye Ojo, Joy Stephen Amusan, Opeoluwa Iwaloye, Uwemedimo Friday Ekpo

**Affiliations:** 1 Department of Microbiology, College of Biosciences, Federal University of Agriculture, Abeokuta, Nigeria; 2 Department of Pediatrics, Federal Medical Center, Abeokuta, Nigeria; 3 Department of Public Health, College of Health Sciences, Osun State University, Osogbo, Nigeria; 4 Department of Pure and Applied Zoology, College of Biosciences, Federal University of Agriculture, Abeokuta, Nigeria

**Keywords:** Traditional Birth Attendants, Clean Delivery kits, Puerperal Fever, Neonatal Infection, WASH, Nigeria

## Abstract

**Background:**

In resource-poor settings, perinatal infections contribute significantly to maternal and neonatal deaths, and the use of clean delivery kits (CDKs) has been proposed as a tool to reduce the risk of infection-related deaths. This study aims to assess the acceptability and effectiveness of CDKs in preventing infections in deliveries attended by traditional birth attendants (TBAs) in Abeokuta, Nigeria.

**Methods:**

The study was a cluster-randomized trial with 67 birth centres/clusters, 453 births/mothers, and 457 babies randomized to intervention or control arms; intervention involved supplementation of delivery with JANMA CDKs. Interviews were conducted at the birth homes, and the primary outcomes were neonatal infection and puerperal fever. The association between infection and perinatal risk factors was tested using the Chi-square and Fisher’s exact tests.

**Results:**

CDKs were well accepted by TBAs. The incidence of puerperal fever and neonatal infection was 1.1% and 11.2%, respectively. Concurrent infection was found in 1 (0.22%) of the mother-neonate pair. There was no significant association between any of the sociodemographic factors and infection for both mothers and neonates. PROM and prolonged labour were significantly associated with puerperal infection. All mothers with puerperal fever were from the control group. Compared to the control group, the relative risk of puerperal infection and neonatal infection in the intervention group was 0.08 (0.004 –1.35, p = 0.079) and 0.64 (0.37 to 1.1, p = 0.10), respectively.

**Conclusion:**

CDKs hold promising results in attenuating maternal infections in resource-poor settings. Larger studies with greater statistical power are required to establish statistically reliable information.

## Background

Although remarkable progress has been made in reducing neonatal and maternal mortality in the last two decades, greater improvements targeting all-cause mortality are needed to meet the Sustainable Development Goals for further reduction of maternal and newborn deaths [1]. Daily, an estimated 7,000 newborns and 810 mothers are lost to preventable deaths related to pregnancy and childbirth [2, 3], with a large proportion of these deaths occurring in low- and middle-income countries (LMICs) [4]. With an estimated 700 neonatal deaths [5] and 145 maternal deaths each day [6], Nigeria is one of the countries in the world with an unacceptably high rate of maternal and neonatal mortality.

Infectious diseases contribute significantly to deaths in mothers and neonates, accounting for about 11% and 30% of global maternal and neonatal deaths, respectively [7, 8]. Deaths from infection occur primarily through sepsis [9] and are most common in poor resource settings [7, 8]. They also reveal important health determinants as well as underlying issues precluding quality care, including infrastructural constraints [10]. Several lines of evidence have shown that improved water, sanitation, and hygiene (WASH) services are indispensable in delivering quality care to mothers and babies, improving their health and well-being, and averting preventable death [11]. Important recommendations for a clean delivery, referred to as the ‘six cleans of delivery,’ have been outlined by the WHO and are significantly associated with reduced incidences of infection during delivery [12].

Despite these known benefits, infrastructures needed to provide adequate WASH conditions are often insufficient, making clean delivery practices unattainable for mothers and their birthing companions [11]. Reports from low- as well as middle-income countries show that WASH services are absent in many healthcare facilities [13]. These gaps extend to delivery and newborn care environments [14], resulting in an increased risk of healthcare-associated infections for mothers and their babies [15]. This is further complicated by the low rates of skilled birth attendance in several regions of the world. While improving skilled attendance at births, many sub-Saharan African countries rely on traditional birth attendants (TBAs) as “interim” partners providing maternal and infant health care in rural areas as well as poor areas of cities. Thus, a significant number of births are handled by TBAs [16] in the absence of clean delivery tools, along with a high risk of infection [17].

Some strategies, including clean delivery kits (CDKs), have been put in place to facilitate clean birth and postnatal care practices [12]. The WHO recommends disposable CDKs as an avenue for providing delivery supplies that ensure hygienic birth conditions and cord care in low-resource settings [12]. Due to their low costs, CDKs are regarded as a very cost-effective means of preventing infections and most likely benefit the poorest families. Although CDKs hold the potential to reduce maternal-neonatal infections, there is a paucity of evidence on their uptake and effectiveness in preventing maternal-neonatal infections among unskilled providers of maternal services in resource-poor areas. Also, evidence for the effectiveness of CDKs has been investigated along with other interventions, making it difficult to interpret the results in the context of CDKs alone. Hence, this study aims to determine the acceptability and effectiveness of single-use CDKs in preventing infections in deliveries attended to by TBAs in Abeokuta, southwest Nigeria.

## Materials and Methods

### Study Location

**Plate 1:**
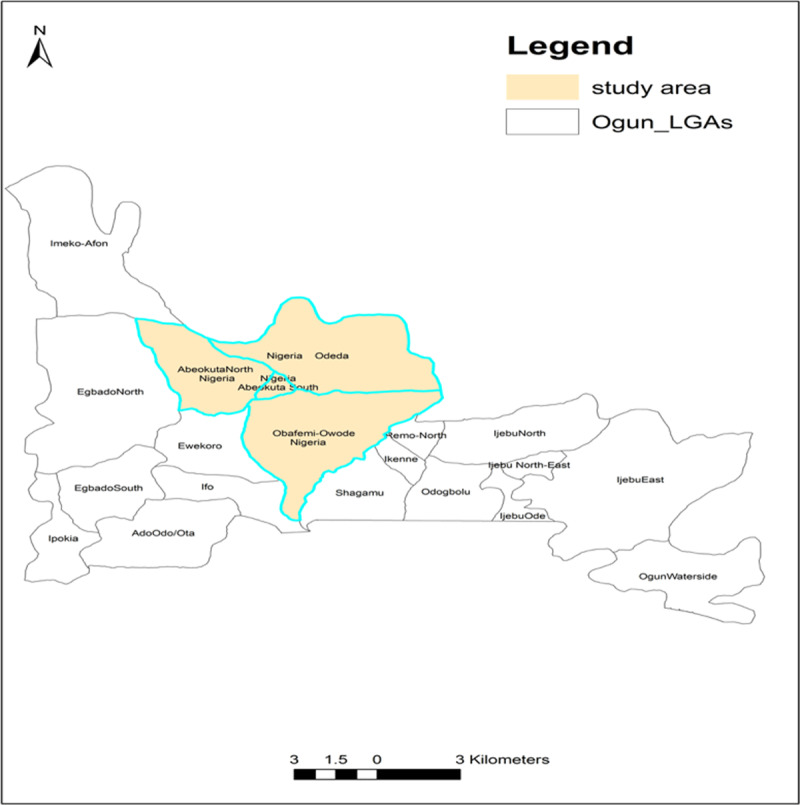
Map of Study Area.

The study was set up in Abeokuta, the largest city in Ogun State, between January 2017 and April 2019 as part of a study evaluating water, sanitation, and hygiene (WASH) conditions in traditional birth centers. The city covers Abeokuta South and some parts of Abeokuta North, Odeda, and Obafemi Owode Local Government Areas (Plate 1). The city is served by a few government hospitals, private hospitals, primary healthcare centres, and scores of community, herbal, and traditional practitioners. TBAs are commonly found in low-income urban areas of the town, where they provide perinatal care to pregnant women. They are recognized as part of the healthcare system and work with the state government through their TBA Association. They are integrated into the state’s primary health care and regulated by the Primary Health Care Development Board.

### Study Design

The study was a cluster-randomized trial, with each birth attendant/centre representing a cluster. The traditional birth attendants/centres were identified through the Traditional Birth Attendant Association. Participating clusters were selected randomly from those registered with the association. The inclusion criteria included rendering perinatal services within Abeokuta and willingness to enroll in the study. Sixty-seven TBAs/clusters were enrolled in the study. Each cluster was randomized to an intervention or control arm. The randomization/allocation sequence from 1 to 100 (based on registration details provided by the TBA Association) generated 50 control and 50 intervention arms. The allocation was adequately concealed using opaque, sealed envelopes. During recruitment, 32 control and 35 intervention arms gave consent and were enrolled. Traditional birth attendants in the intervention clusters received birth kits and were taken through the content of the kit and its use, while those in the control group maintained their routine perinatal care.

### Sample Size

A total of 67 birth centres, 457 neonates, and 453 mothers were recruited into the study. The intervention group consisted of 32 birth centres that enrolled 201 mothers and 203 neonates, while the control group included 35 birth centres enrolling 252 mothers and 254 neonates.

### Ethical Clearance and Informed Consent

Ethical clearance for the study was obtained from the State Primary Health Care Development Board as well as the National Health Research Ethics Committee (through the Federal Medical Centre, Abeokuta). Informed written consent was also obtained from the mothers and permission to conduct the study from the TBAs/birth centres.

### Clean Delivery Kits

**Plate 2:**
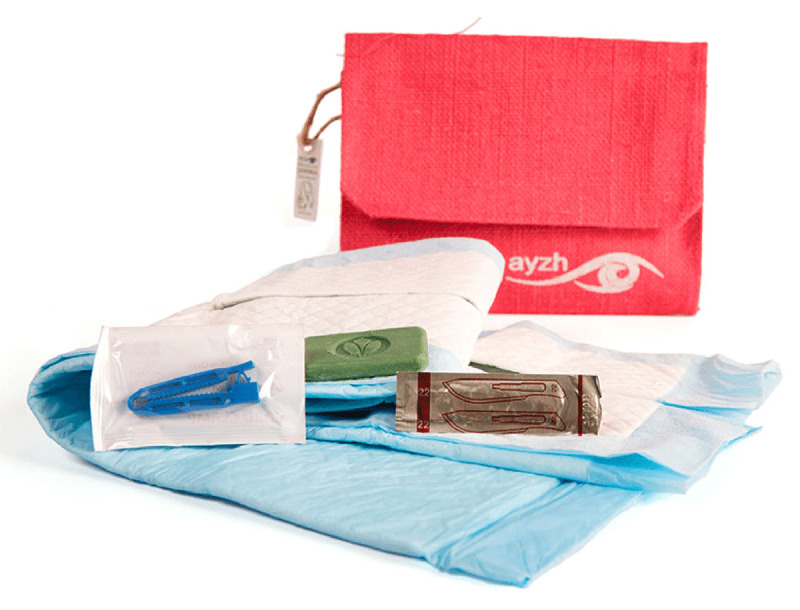
Components of Clean Delivery Kit.

Five hundred JANMA CDKs were provided by AYZH, a for-profit social enterprise, at no cost for this study. Each CDK contained ten items, including soap, gloves, a blood-absorbing underpad, blade, a cord clamp, soft cotton cloth, a chlorhexidine tube, a sanitary pad, pictorial instructions, and a jute purse (Plate 2).

### Data Collection

Research assistants who were trained on the study questionnaire and research ethics conducted interviews within the premises of the traditional birth homes (TBHs) in Yoruba, the local dialect in the study setting.

### Traditional Birth Attendants (TBAs)

Pilot administration of the questionnaire developed for the study was carried out in a small community in Ado-Odo Ota, a Local Government Area in the State to test and refine the questionnaire. Following this pilot, interviews were conducted at the TBHs using the pretested, interviewer-administered questionnaire. The interviews were designed to collect information on sociodemographic characteristics and the scope of TBA practices. To assess CDK use and acceptability, in-depth interviews were conducted within 3 days of delivery and kit use at TBHs. CDK acceptability was based on the following contexts: perceived need, kit components, cultural and religious considerations, operational considerations, and economic considerations.

### Maternal and Neonatal Data

Maternal and neonatal data were obtained using an interviewer-administered questionnaire. Data included:

- Sociodemographic characteristics of mothers;
- Neonatal details (sex, age, and estimated gestational age (EGA)). EGA was based on maternal last menstrual dates or early scans where this was available.
- Pregnancy and perinatal events (parity, maternal perinatal fever, urinary tract infection, excessive vaginal discharge, place of delivery, prolonged rupture of membrane, and labour duration); and
- Maternal and neonatal clinical details (temperature and clinical manifestations of sepsis). Socio-economic status was assessed as previously described by Ogunlesi and Ogunfowora, 2015 [18].

### Maternal and Neonatal Outcomes

The primary outcomes were neonatal infection and puerperal fever. Visits were made 24–72 hours after birth to assess the mothers and their babies, and signs of infection were recorded. Criteria for infection in neonates were defined according to the algorithm recommended by the World Health Organization’s (WHO) Young Infant Study Group. This simple clinical criteria was developed by the group to identify neonates with signs of severe bacterial infection [19]. Infection in the neonates was defined according to this algorithm as the presence of at least one of these seven signs during assessment: (i) history of or observed convulsion; (ii) respiratory rate of ≥60 per minute; (iii) poor or reduced feeding; (iv). severe chest indrawing; (v) hypothermia/temperature ≤ 35.5 °C; (vi) hyperthermia/temperature ≥ 37.5 °C hypothermia/temperature ≤ 35.5 °C; (vii) lack of spontaneous movement [19]. A preliminary diagnosis of puerperal fever was made as previously described by a WHO technical working group as infection of the genital tract occurring at any time between the onset of rupture of membranes or labour and 42 days postpartum in which two or more of the following are present: pelvic pain, fever, abnormal vaginal discharge, abnormal or foul odour, discharge, or delay in uterine involution [20].

### Statistical Analysis of Data

The data from the study was analysed using the Statistical Package for Social Sciences (SPSS) software (version 25.0). The results were presented as frequencies and percentages for categorical variables. The association between infection in mothers and neonates and perinatal risk factors was tested using the Chi-square and Fisher’s exact tests, with statistical significance set at P < 0.05. The relative risk (RR) of infection was calculated with a 95% confidence interval (CI) using the electronic version of the MedCalc Relative Risk Statistical Calculation Software.

## Results

### Study Participants

- Traditional Birth Centres/Attendants
  Sixty-seven TBAs were recruited into the study. About one-fifth of the TBAs offered perinatal services only, while other TBAs provided other health services. TBAs were mostly older women with no formal education (Table 1).
- Mothers and Neonates
  More than half (56%) of the women in the study were 21–30 years of age, and 60% were multiparous. Most of the mothers (96%) were classified as belonging to the lower socio-economic class. There was an approximately 1:1 male-to-female ratio among the neonates. More than half (53%) of the neonates were between 24 and 48 hours old.

**Table 1:** Sociodemographic Characteristics of the Traditional Birth Attendants in the Study.


FEATURES	VARIABLES	FREQUENCY (N)	PERCENTAGES (%)

Sex	Female	45	82.1

	Male	12	17.9

Age	20–30	05	07.5

	30–40	15	22.4

	40–50	26	38.8

	>50	21	31.3

Service provided	General	51	76.1

	Perinatal	16	23.9

Average number of Delivery	1–5	50	73.1

	5–10	09	13.4

	11–15	07	10.4

	16–20	01	1.5

	>20	03	4.5

Study Group	Intervention	32	47.8

	Control	35	52.2


### Puerperal Fever and Neonatal Infection

- Clinical Symptoms in Mothers and Neonates
  Hyperthermia and hypothermia were the most common symptoms, occurring in 32 (7%) and 15 (3.3%) of the neonates, respectively. The least common symptom was lethargy in less than 1% of the neonates. Lower abdominal pain was reported by 8% of the mothers, and this was the most common symptom (Table 2).
- Risk Factors
  The risk factors for infection in the mothers and their neonates are shown in Tables 3 and 4. Premature rupture of membranes was found in 31 (6.8%) of the mothers. More than half (52.5%) of the neonates studied were preterm, and nine (1.8%) were born after prolonged labour.
- Incidence of neonatal infection and puerperal fever
  Of the 453 women in the study, five (1.1%) had symptoms and/or signs indicating puerperal fever. The total number of live births was 457. Fifty-one (11.2%) of the neonates had symptoms and/or signs indicating neonatal infection. Concurrent infection was found in 1 (0.22%) of the mother-neonate pair.
- Association between Risk Factors and Infection
  Two risk factors found to be statistically associated with puerperal fever in mothers were prolonged rupture of membranes and prolonged labour (p < 0.001, p = 0.003, respectively). None of the socio-demographic factors, signs or symptoms, or maternal risk factors had a statistically significant association with neonatal infections (Tables 3 and 4).
- Effectiveness of CDK Use
  All mothers with puerperal fever belonged to the non-intervention group. There was a 2% incidence rate of puerperal fever in this group compared to the CDK group (0%). There were also more neonatal infections in the control group (12.9%) compared to those who used CDK (8.2%). However, statistical analysis showed that the association between kit use and maternal or neonatal infections was not significant, with p-values of 0.123 and 0.101, respectively (Tables 3 and 4). Compared to the control group, the relative risk of puerperal infection and neonatal infection in the intervention group was 0.08 (0.004–1.35, p = 0.079) and 0.64 (0.37 to 1.1, p = 0.10), respectively (Table 5).

**Table 2:** Signs and Symptoms in Mothers and Neonates.


	SIGNS/SYMPTOMS	FREQUENCY (N)	PERCENTAGES (%)

Maternal	Hyperthermia	12	2.4

	Lower Abdominal Pain	23	7.0

	Lower Abdominal Tenderness	21	6.8

Neonatal	Poor Feeding Ability	08	1.7

	Poor spontaneous Movement	03	52.9

	Hyperthermia	32	7.0

	Hypothermia	15	3.3


**Table 3:** Association Between Puerperal Fever, Socio-demographic Factors, Perinatal Risk Factors and Kit Use.


VARIABLE	INFECTION		STATISTICS

	ABSENT	PRESENT	

**Maternal Age**			

<20	69(95.8)	3(4.2)	X^2^ = 5.146

21–40	379(99.5)	2(0.5)	P = 0.076

>40	3(100)	0(0)	

**Marital status**			

Married	433(98.9)	5(1.1)	X^2^ = 0.000

Single	18(100)	0(0)	P = 1.000

**Socioeconomic class**			

Low	432(98.9)	5(1.1)	X^2^ = 0.000

Middle	19(100)	0(0)	P = 1.000

**Parity**			

Primiparous	138(97.2)	4(2.8)	X^2^ = 5.324

Multiparous	282(99.6)	1(0.4)	P = 0.070

Grand-multiparous	30(100)	0(0)	

**PROM**			

No	433(99.5)	2(0.5)	X^2^ = 23.713

Yes	18(85.7)	3(14.3)	**P < 0.001**

**Prolonged labor**			

No	445(99.3)	3(0.7)	X^2^ = 23.400

Yes	6(75)	2(25)	**P = 0.0026**

**Preterm Birth**			

No	249(99.2)	2(0.8)	X^2^ = 0.052

Yes	202(98.5)	3(1.5)	P = 0.820

**Kit Use**			

Yes	250(98)	0(0)	X^2^ = 2.382

No	201(100)	5(2)	P = 0.123


**Table 4:** Association Between Neonatal Infection, Socio-demographic Factors, Perinatal Risk Factors and Kit Use.


VARIABLE	INFECTION		STATISTICS

	ABSENT	PRESENT	

**Sex**			

Female	199(88.4)	26(11.6)	X^2^ = 0.506

Male	210(90.9)	21(9.1)	P = 0.477

**Age (hours)**			

0–24	130(91.5)	12(8.5)	X^2^ = 1.133

25–48	211(88.3)	28(11.7)	P = 0.568

49–72	68(90.7)	7(9.3)	

**Maternal Socioeconomic class**			

Low	391(89.5)	46(10.5)	X^2^ = 0.125

Middle	18(94.7)	1(5.3)	P = 0.724

**Maternal Parity**			

Primiparous	131(92.3)	11(7.7)	X^2^ = 1.664

Multiparous	251(88.7)	32(11.3)	P = 0.435

Grand-multiparous	26(86.7)	4(13.3)	

**PROM**			

No	390(89.7)	45(10.3)	X^2^ = 0.000

Yes	19(90.5)	2(9.5)	P = 1.000

**Prolonged labor**			

No	402(89.7)	46(10.3)	X^2^ = 0.000

Yes	7(87.5)	1(12.5)	P = 1.000

**Preterm Birth**			

No	228(90.8)	23(9.2)	X^2^ = 0.539

Yes	181(88.3)	24(11.7)	P = 0.463

**Kit Use**			

Yes	234(91.8)	21(8.2)	X^2^ = 2.686

No	175(87.1)	26(12.9)	P = 0.101


**Table 5:** Effectiveness of Clean Delivery Kits in Reducing the Risk of Infection in Mothers and their Neonates.


	VARIABLE	INFECTION		RELATIVE RISK	P-VALUE

		ABSENT	PRESENT		

**Mothers**	**Kit Use**			0.08 (0.004–1.35)	0.079

	Yes	250(98)	0(0)		

	No	201(100)	5(2)		

**Neonates**	**Kit Use**			0.64 (0.37–1.1)	0.10

	Yes	234(91.8)	21(8.2)		

	No	175(87.1)	26(12.9)		


### Acceptability of CDKs

CDK acceptability was based on the following contexts:

- Perceived Need
  Most TBAs were aware of the need for hygienic deliveries and some of the inherent dangers associated with unhygienic births, especially with regard to HIV transmission, cord infections, and tetanus in mothers and newborns.
- Kit Components
  All components of the kit were acceptable to TBAs except for the cord clamp, which TBAs were either unaccustomed to or had reservations about. This observation was made during study sensitization, and sterile cord ties were added to the birth kits.
- Cultural and Religious Considerations
  There was no cultural or religious bias against the use of kit components. CDKs were acceptable to TBAs from diverse religious and cultural/tribal groups.
- Operational considerations
  The CDKs conformed well with TBA practices, which they found useful for each item. The function of each item was understood by the TBAs. A noteworthy attribute of the CDK was convenience. All items needed for a clean delivery were in a single purse.
- Economic considerations
  Although TBAs highlighted CDK’s convenience and hygiene promotion, many expressed concerns about the additional cost to their clients, who may be unable to pay extra costs for the kits. At the time of the study, the cost of each kit was about $2.20 when kit materials were sourced from the local market. There were no commercially available clean delivery kits on the market. The content of delivery kits varied greatly between hospitals within the study area, and many routinely provided clean delivery items as part of delivery care.

## Discussion

The effectiveness of CDKs has been investigated in poor-resource settings, mainly in light of home births along with other interventions. This has made it difficult to interpret results on effectiveness. In this study, CDKs were handed directly to TBAs in low-income areas of the state capital for their use during deliveries. This ensured that a TBA was identified as a kit user and that the only intervention received during the study was the CDK.

As with most studies, TBAs were mostly older, female, community-based providers of maternal care without any education or formal training. The large number of TBAs in the study setting, as well as the births involved in the study, affirm that TBAs still account for a remarkable proportion of births, even in cities in developing countries. Although the drive for increased skilled birth attendance remains a top priority, the large number of births covered by TBAs during this transition to skilled birth attendance makes continuous engagement with them a critical component of maternal care [21].

The incidence rate of puerperal infection (1.1%) in the study is similar to other reports from Nigeria and other sub-Saharan countries [22, 23], with higher rates reported in similar settings [24, 25]. While the diagnosis of puerperal infection covers the first 42 days postpartum [20], mothers in this study were evaluated only within the first three days postpartum. Therefore, the incidence of 1.1% may poorly reflect the actual cases of puerperal infection among the study population. Prolonged labour and rupture of membranes were found to be statistically associated with puerperal fever. Anaemia in pregnancy, premature rupture of membranes, frequent vaginal examination during labour, and prolonged labour are other risk factors that have been identified with puerperal infections in Nigeria [26,27].

The incidence of neonatal infection (11.2%) was comparable to similar studies in Nigeria and Sub-Saharan Africa [28,29]. Incidences reported in hospital births are unsurprisingly lower. An earlier study in a tertiary hospital located in the study area reported an incidence of 2.8/100 live births for culture-proven sepsis. The most frequent risk factors for neonatal infection in this study are similar to those described in other studies. A review of studies across Nigeria as well as in other developing countries identified prematurity [30,31,32,33] PROM [30, 32,33,34], maternal pyrexia [32, 34, 35], low birthweight [31, 36,37,38], and difficulties at delivery (including birth asphyxia or obstructed labour) [35, 39] as risk factors for neonatal sepsis. The high rate of preterm births, a major risk factor for early-onset sepsis, observed in the study is noteworthy, as about half of the neonates were born preterm. A possible explanation for these high rates of preterm births is the use of herbs across these birth homes as well as the poor socio-economic condition of the mothers [40].

The differences in rates of reduction in neonatal and maternal infection between intervention and control groups were not statistically significant. The statistical power for the analysis of the effect of CDK on the outcomes in this study could be limited by the small size of CDK users recruited, as these outcomes are either rare (puerperal infection) or present non-specific signs and symptoms (neonatal infections). Studies in other LMICs have reported that the use of CDK during birth is associated with a reduced risk of infection and improvements in neonatal outcomes [9, 41]. Reductions in early newborn mortality with CDK use are reported in facility deliveries as well as home settings [42], The reduction in cases of puerperal infections is consistent with findings from other studies on CDK use in homes as well as facilities [43, 44]. Other studies have reported an increased risk of adverse outcomes with CDK use.

A recent study in Northern Nigeria found no association between the use of CDKs and reduced maternal or neonatal morbidity and suggests the possibility of increased risk of adverse outcomes when CDKs are made available to women outside the context of a structured health system [41]. A major difference in this study may be the distribution of kits to TBAs rather than pregnant women. TBAs in this setting are recognized by the Primary Health Care of the State, and their practices are regulated by the Primary Health Care Development Board. The board also provides training and other forms of support for the TBAs. Compliance with kit use was high among this population and may not be reflective of TBAs in low-resource settings in general.

The cost of local assembly of CDK ($2.20) compared to the lowest average patient cost of treating blood stream infection in neonates in Africa ($20.90) [45] buttresses the cost-effectiveness of a clean delivery kit in preventing infections in these settings. Assessment of clean delivery kit acceptability among the TBAs revealed that the kits were perceived as acceptable, appropriate, and essential items in ensuring clean deliveries. However, financial constraints may preclude its routine use by the TBAs during delivery.

## Conclusion

Results from this study support the use of CDKs in encouraging clean deliveries among traditional birth attendants and reducing infection in mothers and babies who are yet to be reached by skilled birth workers. Results from this study may be useful in similar settings characterized by poor rates of skilled birth attendance and maternal-neonatal birth outcomes, especially in rural areas where TBAs continue to lead maternal and infant care. Although the kits are low-cost interventions, concerns expressed by TBAs about kit cost suggest uptake may still be limited by financial constraints.

### Limitations

There were some noteworthy limitations to this study. The diagnosis of infection in this study was based on clinical signs/symptoms and did not involve the use of blood cultures, the gold standard, or any other laboratory findings. Likewise, the WHO Young Infant Study algorithm used in the diagnosis of neonatal infections presents with high sensitivity and low specificity due to its overlap with other conditions, including birth asphyxia and prematurity. The case of prematurity is of particular interest, as more than half of our study population was preterm. This low specificity with neonatal infections as well as the rare outcome of puerperal infections may also have impacted our analysis, limiting the statistical power.

This study also focused on infections within the first 72 hours of life, and our findings do not cover the entire spectrum of early-onset maternal or neonatal infections. Due to the dependence on TBAs’ recall of kit use, a possible recall bias could impact the true reflection of events surrounding delivery. Nonetheless, this should be minimal, as the recall was done within 72 hours of birth.
